# Left atrial dynamics during in-scanner exercise: a CMR myocardial feature tracking study

**DOI:** 10.1186/1532-429X-18-S1-O80

**Published:** 2016-01-27

**Authors:** Vera-Christine Stahnke, Johannes T Kowallick, Eckhart Thassilo von Loesch, Michael Steinmetz, Wieland Staab, Jan M Sohns, Martin Fasshauer, Antonia Zapf, Joachim Lotz, Andreas Schuster, Christina Unterberg-Buchwald

**Affiliations:** 1 Institute of Diagnostic and Interventional Radiology, Goettingen, Germany; 2 Dept. of Pediatric Cardiology, University Clinic Goettingen, Goettingen, Germany; 3 Institute of Medical Statistics, University Goettingen, Goettingen, Germany; 4 Dept. of Cardiology and Pneumology, University Clinic Goettingen, Goettingen, Germany

## Background

LA functional abnormalities are increasingly recognized to have the potential to predict the outcome in a variety of cardiovascular disease states. However, little is known about LA dynamics during physical exercise. The objective of this study was to examine LA function using cardiovascular magnetic resonance feature tracking (CMR-FT) in volunteers during physiological exercise with a supine in-scanner ergometer.

## Methods

15 healthy volunteers were enrolled for supine cycle ergometry on the scanner table using a MR-compatible ergometer (Lode,The Netherlands). Imaging was performed at 3T (Siemens Skyra). Standard 2- and 4-chamber steady state free precession (SSFP) cine images were acquired at rest and after 3 minutes of cycling at 50W and 100W during a short break of cycling to minimize motion artifacts. LA CMR-FT (TomTec Imaging Systems, Germany) was performed in 2-chamber view. Three aspects of LA function were analyzed using longitudinal strain and strain rate parameters:(1)LA reservoir function (total strain [Es], peak positive strain rate [SRs]);(2)LA conduit function (passive strain [Ee], early negative strain rate [SRe]) and (3)LA contractile booster pump function (active strain [Ea], late negative strain rate [SRa]). LA phasic volumes (volume at left ventricular end-systole/maximum left atrial volume [V.max], volume before atrial contraction/left atrial volume prior to left atrial contraction [V.p-ac] and minimal volume at ventricular end-diastole/minimum left atrial volume [V.min]) were quantified from 2- and 4-chamber views using the biplane-area length method. Left atrial emptying fractions were calculated: LAEF total (V.max-V.min) × 100 / V.max; LAEF passive (V.max-V.p-ac) × 100 / V.max and LAEF booster (V.p-ac-V. min) × 100 / V.p-ac.

## Results

LA deformation indexes and phasic volumes are summarized in Tab.1. LA CMR-FT and LA volumetry were successfully performed in all subjects at rest. After 50W and 100W exercise, cine SSPF images of one and three volunteers had to be excluded due to considerable breathing artifacts, respectively. Phasic volumes (both long axis with good quality) were undetectable in one/two different volunteers at 50W/100W.

LA strain parameters showed a non-significant trend to increase between rest and exercise, corresponding to reservoir and conduit function. Moreover, all strain rate parameters increased significantly between rest and exercise. No additional increase in strain rate parameters was detected between 50 and 100W. Maximal LA volumes increased with exercise but neither passive nor booster volume fractions changed significantly from rest to exercise.

## Conclusions

CMR-FT derived LA function analysis is feasible during dynamic exercise stress using a supine MR-compatible ergometer. LA strain rate parameters may be most sensitive to detect physiological responses to exercise. The future potential of LA CMR-FT during in-scanner exercise for early detection of LA functional abnormalities will need to be addressed in further patient studies.

**Table 1:** Left atrial volumes, deformation parameters and heart rate (HR) at rest and during supine exercise with 50 and 100 Watts (W); mean ± standard deviation.

	Rest	50W	100W	Rest vs. 50W	Rest vs. 100W	50W vs. 100W
				P value		
**HR [beats/min]**	59.6 ± 12.4	93.8 ± 14.8	116.8 ± 12.3	**< 0.001**	**< 0.001**	**< 0.001**
**V.max [ml]**	65.7 ± 18.7	65.4 ± 21.0	68.4 ± 23.5	0.903	0.94	0.979
**V.p-ac [ml]**	35.9 ± 11.3	31.2 ± 17.8	31.6 ± 13.7	0.375	0.39	0.742
**V.min [ml]**	26.0 ± 11.6	16.8 ± 8.6	19.7 ± 9.6	**0.014**	0.113	0.45
**LAEF total [%]**	61 ± 9	75 ± 7	71 ± 8	**< 0.001**	**0.013**	0.23
**LAEF passive [%]**	44 ± 14	55 ± 13	54 ± 9	0.059	0.085	0.976
**LAEF booster [%]**	27 ± 25	43 ± 17	37 ± 17	0.079	0.248	0.475
**LA Reservoir Function**						
**Es [%]**	33.3 ± 14.1	41.8 ± 13.5	40.5 ± 14.5	0.063	0.089	0.487
**SRs [s-1]**	1.3 ± 0.5	1.9 ± 0.6	2.0 ± 0.7	**0.003**	**< 0.001**	0.93
**LA Conduit Function**						
**Ee [%]**	21.1 ± 11.2	26.6 ± 9.5	25 ± 12.4	0.052	0.21	0.576
**SRe [s-1]**	-1.0 ± 0.3	-1.6 ± 0.5	-1.6 ± 1.0	**< 0.001**	**0.024**	0.979
**LA Booster Pump Function**						
**Ea [%]**	12.2 ± 6.2	15.2 ± 7.7	15.5 ± 4.6	0.281	0.178	0.611
**SRa [s-1]**	-0.9 ± 0.4	-1.5 ± 0.7	-1.8 ± 0.9	**0.008**	**0.002**	0.73

HR, heart rate; V.max., maximum left atrial volume; V.p-ac, left atrial volume prior to left atrial contraction; V.min., minimum left atrial volume; LAEF, left atrial emptying fraction; E, strain; SR, strain rate. Bold p values indicate a significance level < 0.05 as determined by Student's paired t-test.

